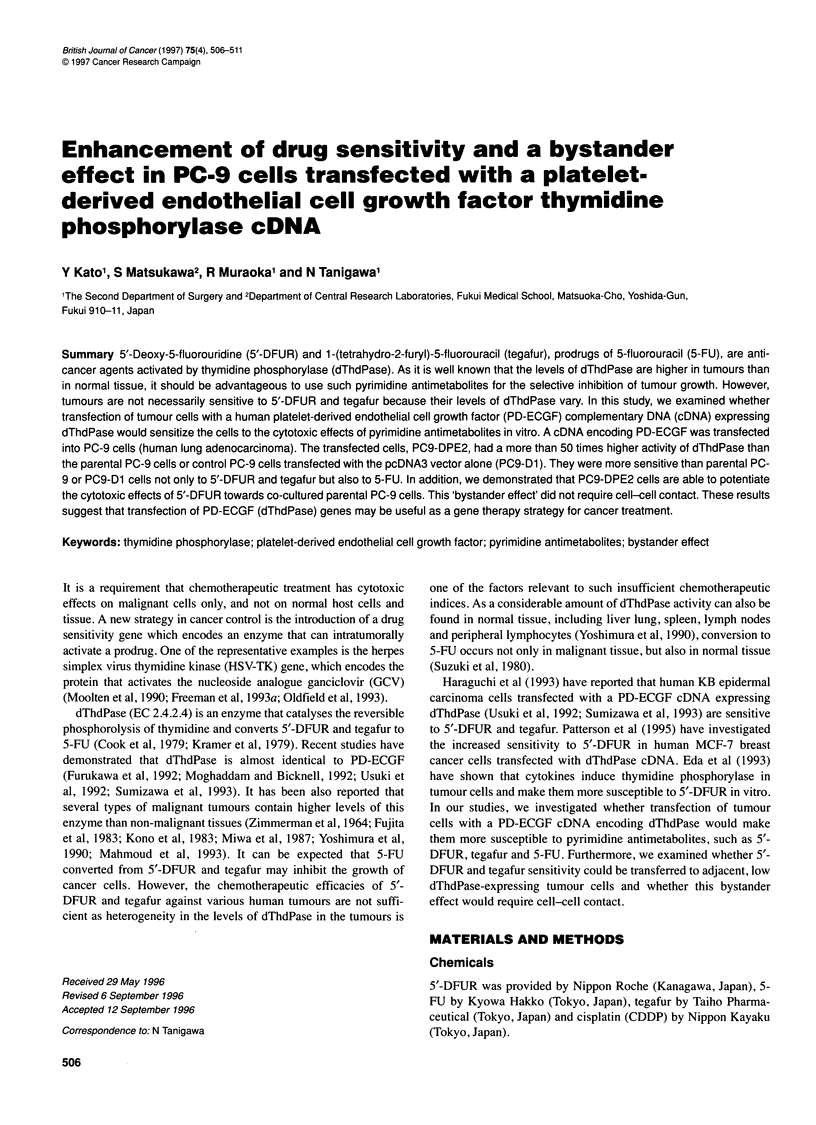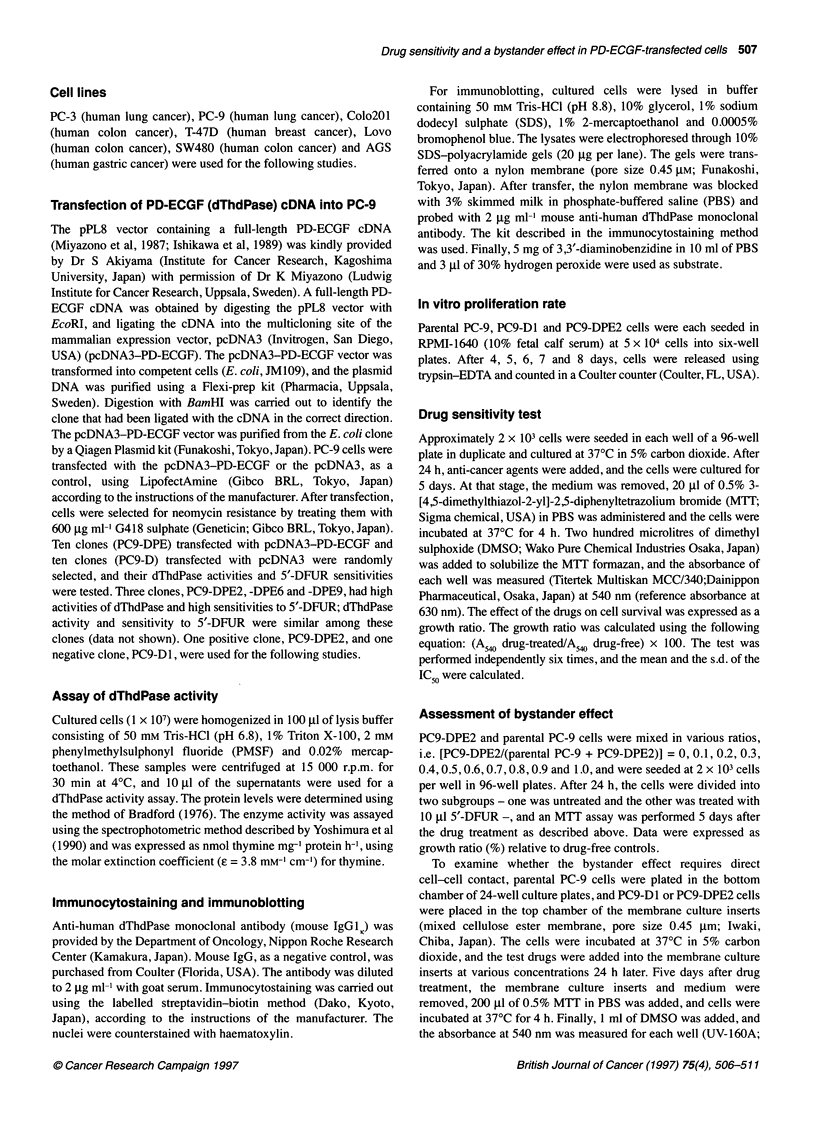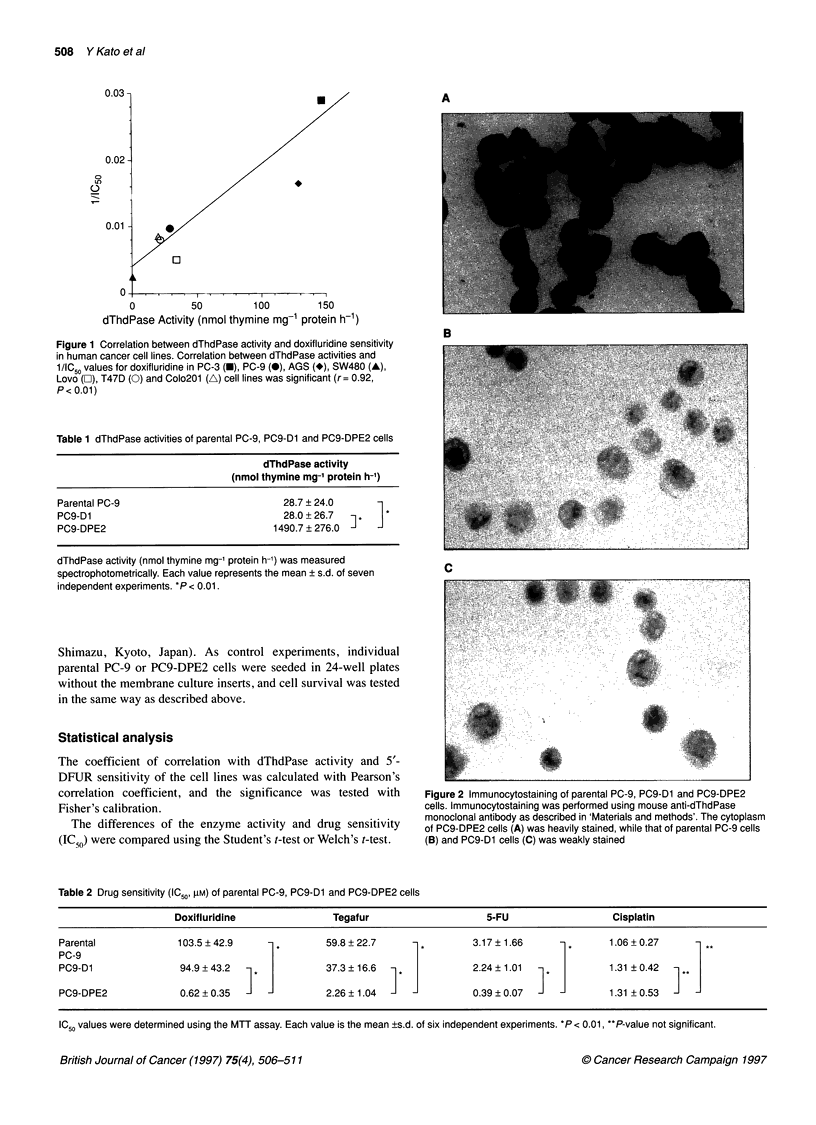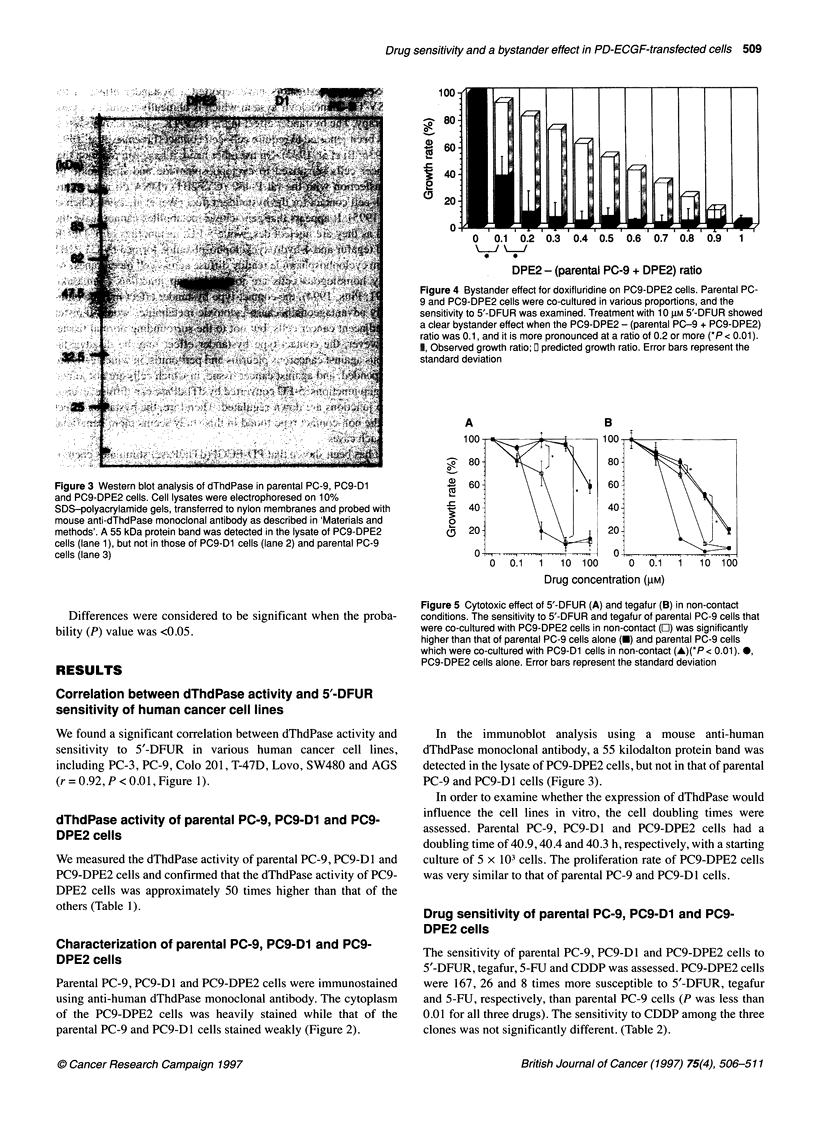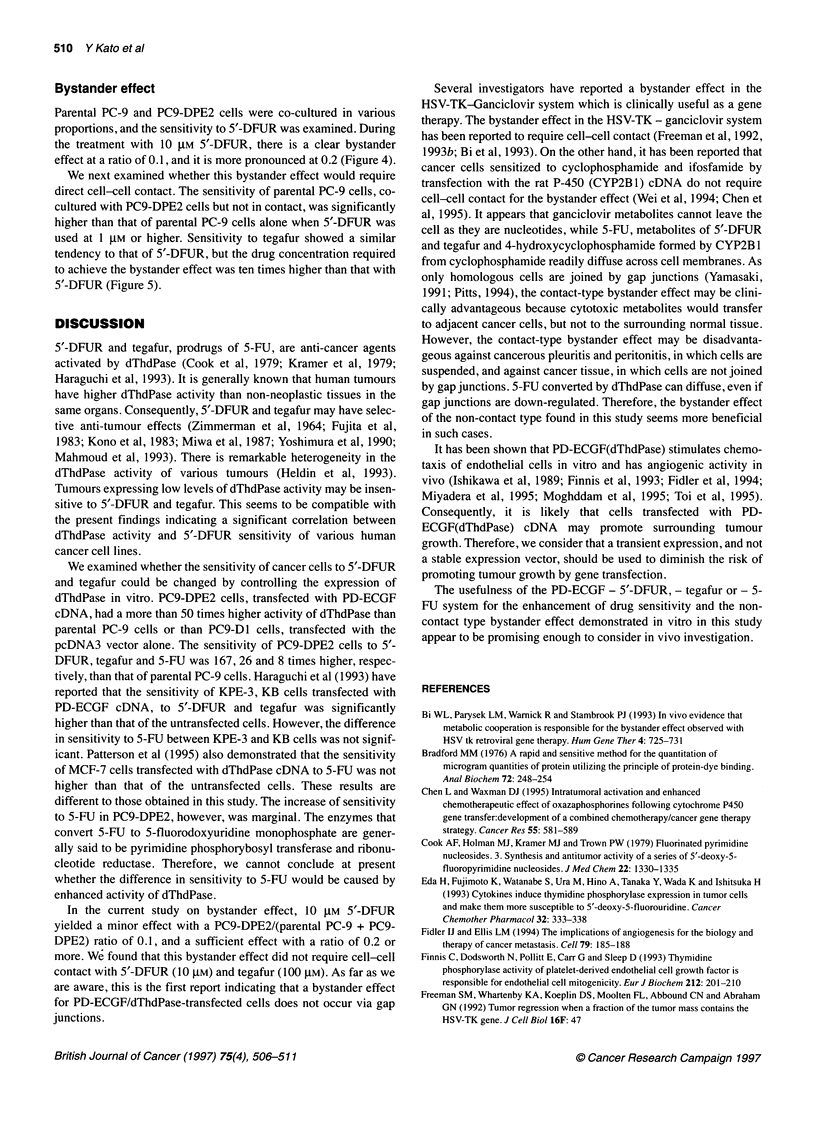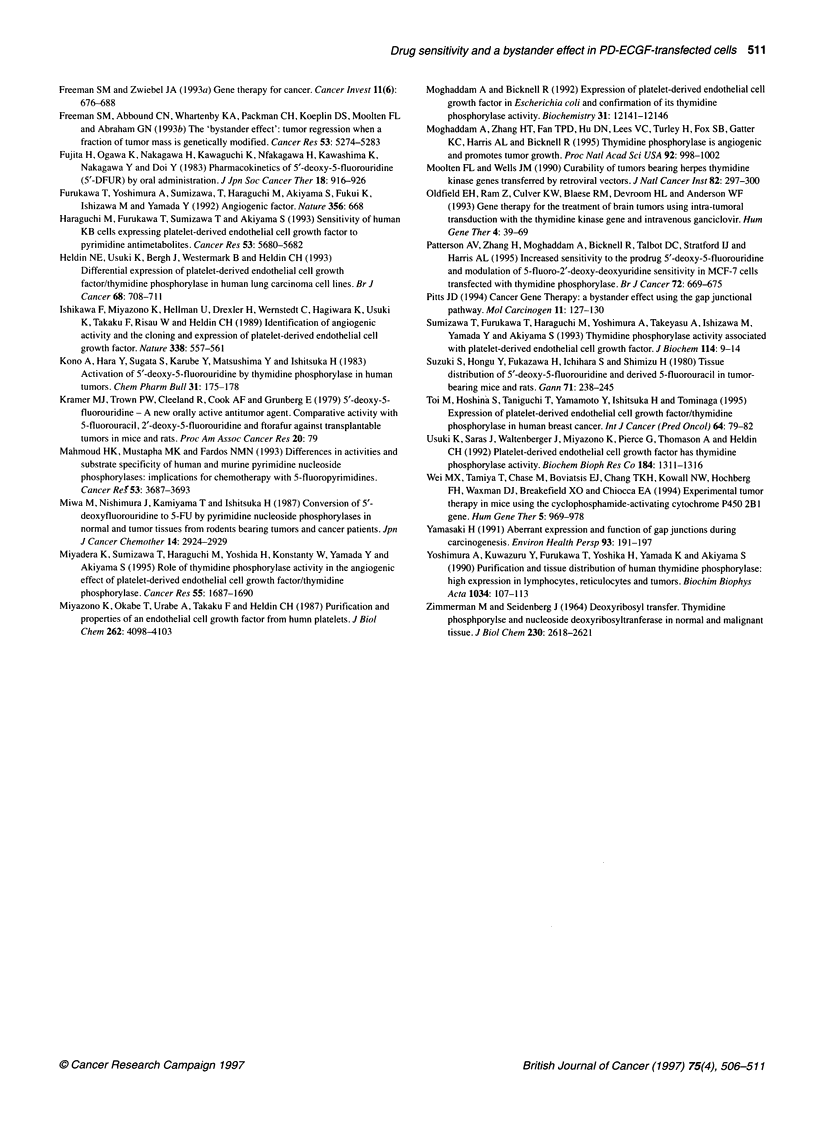# Enhancement of drug sensitivity and a bystander effect in PC-9 cells transfected with a platelet-derived endothelial cell growth factor thymidine phosphorylase cDNA.

**DOI:** 10.1038/bjc.1997.88

**Published:** 1997

**Authors:** Y. Kato, S. Matsukawa, R. Muraoka, N. Tanigawa

**Affiliations:** Second Department of Surgery, Fukui Medical School, Japan.

## Abstract

**Images:**


British Journal of Cancer (1997) 75(4), 506–511
© 1997 Cancer Research Campaign

### Enhancement of drug sensitivity and a bystander effect in PC-9 cells transfected with a platelet-derived endothelial cell growth factor thymidine phosphorylase cDNA

**Y Kato[1], S Matsukawa[2], R Muraoka[1] and N Tanigawa[1]**

[1]The Second Department of Surgery and [2]Department of Central Research Laboratories, Fukui Medical School, Matsuoka-Cho, Yoshida-Gun, Fukui 910–11, Japan

**Summary** 5′-Deoxy-5-fluorouridine (5′-DFUR) and 1-(tetrahydro-2-furyl)-5-fluorouracil (tegafur), prodrugs of 5-fluorouracil (5-FU), are anti-cancer agents activated by thymidine phosphorylase (dThdPase). As it is well known that the levels of dThdPase are higher in tumours than in normal tissue, it should be advantageous to use such pyrimidine antimetabolites for the selective inhibition of tumour growth. However, tumours are not necessarily sensitive to 5′-DFUR and tegafur because their levels of dThdPase vary. In this study, we examined whether transfection of tumour cells with a human platelet-derived endothelial cell growth factor (PD-ECGF) complementary DNA (cDNA) expressing dThdPase would sensitize the cells to the cytotoxic effects of pyrimidine antimetabolites in vitro. A cDNA encoding PD-ECGF was transfected into PC-9 cells (human lung adenocarcinoma). The transfected cells, PC9-DPE2, had a more than 50 times higher activity of dThdPase than the parental PC-9 cells or control PC-9 cells transfected with the pcDNA3 vector alone (PC9-D1). They were more sensitive than parental PC-9 or PC9-D1 cells not only to 5′-DFUR and tegafur but also to 5-FU. In addition, we demonstrated that PC9-DPE2 cells are able to potentiate the cytotoxic effects of 5′-DFUR towards co-cultured parental PC-9 cells. This 'bystander effect' did not require cell–cell contact. These results suggest that transfection of PD-ECGF (dThdPase) genes may be useful as a gene therapy strategy for cancer treatment.

**Keywords:** thymidine phosphorylase; platelet-derived endothelial cell growth factor; pyrimidine antimetabolites; bystander effect

It is a requirement that chemotherapeutic treatment has cytotoxic effects on malignant cells only, and not on normal host cells and tissue. A new strategy in cancer control is the introduction of a drug sensitivity gene which encodes an enzyme that can intratumorally activate a prodrug. One of the representative examples is the herpes simplex virus thymidine kinase (HSV-TK) gene, which encodes the protein that activates the nucleoside analogue ganciclovir (GCV) (Moolten et al, 1990; Freeman et al, 1993*a*; Oldfield et al, 1993).

dThdPase (EC 2.4.2.4) is an enzyme that catalyses the reversible phosphorolysis of thymidine and converts 5′-DFUR and tegafur to 5-FU (Cook et al, 1979; Kramer et al, 1979). Recent studies have demonstrated that dThdPase is almost identical to PD-ECGF (Furukawa et al, 1992; Moghaddam and Bicknell, 1992; Usuki et al, 1992; Sumizawa et al, 1993). It has been also reported that several types of malignant tumours contain higher levels of this enzyme than non-malignant tissues (Zimmerman et al, 1964; Fujita et al, 1983; Kono et al, 1983; Miwa et al, 1987; Yoshimura et al, 1990; Mahmoud et al, 1993). It can be expected that 5-FU converted from 5′-DFUR and tegafur may inhibit the growth of cancer cells. However, the chemotherapeutic efficacies of 5′-DFUR and tegafur against various human tumours are not sufficient as heterogeneity in the levels of dThdPase in the tumours is one of the factors relevant to such insufficient chemotherapeutic indices. As a considerable amount of dThdPase activity can also be found in normal tissue, including liver lung, spleen, lymph nodes and peripheral lymphocytes (Yoshimura et al, 1990), conversion to 5-FU occurs not only in malignant tissue, but also in normal tissue (Suzuki et al, 1980).

Haraguchi et al (1993) have reported that human KB epidermal carcinoma cells transfected with a PD-ECGF cDNA expressing dThdPase (Usuki et al, 1992; Sumizawa et al, 1993) are sensitive to 5′-DFUR and tegafur. Patterson et al (1995) have investigated the increased sensitivity to 5′-DFUR in human MCF-7 breast cancer cells transfected with dThdPase cDNA. Eda et al (1993) have shown that cytokines induce thymidine phosphorylase in tumour cells and make them more susceptible to 5′-DFUR in vitro. In our studies, we investigated whether transfection of tumour cells with a PD-ECGF cDNA encoding dThdPase would make them more susceptible to pyrimidine antimetabolites, such as 5′-DFUR, tegafur and 5-FU. Furthermore, we examined whether 5′-DFUR and tegafur sensitivity could be transferred to adjacent, low dThdPase-expressing tumour cells and whether this bystander effect would require cell–cell contact.

### MATERIALS AND METHODS

### Chemicals

5′-DFUR was provided by Nippon Roche (Kanagawa, Japan), 5-FU by Kyowa Hakko (Tokyo, Japan), tegafur by Taiho Pharmaceutical (Tokyo, Japan) and cisplatin (CDDP) by Nippon Kayaku (Tokyo, Japan).

*Received 29 May 1996*
*Revised 6 September 1996*
*Accepted 12 September 1996*

*Correspondence to:* N Tanigawa

### Cell lines

PC-3 (human lung cancer), PC-9 (human lung cancer), Colo201 (human colon cancer), T-47D (human breast cancer), Lovo (human colon cancer), SW480 (human colon cancer) and AGS (human gastric cancer) were used for the following studies.

### Transfection of PD-ECGF (dThdPase) cDNA into PC-9

The pPL8 vector containing a full-length PD-ECGF cDNA (Miyazono et al, 1987; Ishikawa et al, 1989) was kindly provided by Dr S Akiyama (Institute for Cancer Research, Kagoshima University, Japan) with permission of Dr K Miyazono (Ludwig Institute for Cancer Research, Uppsala, Sweden). A full-length PD-ECGF cDNA was obtained by digesting the pPL8 vector with *Eco*RI, and ligating the cDNA into the multicloning site of the mammalian expression vector, pcDNA3 (Invitrogen, San Diego, USA) (pcDNA3–PD-ECGF). The pcDNA3–PD-ECGF vector was transformed into competent cells (*E. coli*, JM109), and the plasmid DNA was purified using a Flexi-prep kit (Pharmacia, Uppsala, Sweden). Digestion with *Bam*HI was carried out to identify the clone that had been ligated with the cDNA in the correct direction. The pcDNA3–PD-ECGF vector was purified from the *E. coli* clone by a Qiagen Plasmid kit (Funakoshi, Tokyo, Japan). PC-9 cells were transfected with the pcDNA3–PD-ECGF or the pcDNA3, as a control, using LipofectAmine (Gibco BRL, Tokyo, Japan) according to the instructions of the manufacturer. After transfection, cells were selected for neomycin resistance by treating them with 600 μg ml$^{-1}$ G418 sulphate (Geneticin; Gibco BRL, Tokyo, Japan). Ten clones (PC9-DPE) transfected with pcDNA3–PD-ECGF and ten clones (PC9-D) transfected with pcDNA3 were randomly selected, and their dThdPase activities and 5′-DFUR sensitivities were tested. Three clones, PC9-DPE2, -DPE6 and -DPE9, had high activities of dThdPase and high sensitivities to 5′-DFUR; dThdPase activity and sensitivity to 5′-DFUR were similar among these clones (data not shown). One positive clone, PC9-DPE2, and one negative clone, PC9-D1, were used for the following studies.

### Assay of dThdPase activity

Cultured cells ($1 \times 10^7$) were homogenized in 100 μl of lysis buffer consisting of 50 mM Tris-HCl (pH 6.8), 1% Triton X-100, 2 mM phenylmethylsulphonyl fluoride (PMSF) and 0.02% mercaptoethanol. These samples were centrifuged at 15 000 r.p.m. for 30 min at 4°C, and 10 μl of the supernatants were used for a dThdPase activity assay. The protein levels were determined using the method of Bradford (1976). The enzyme activity was assayed using the spectrophotometric method described by Yoshimura et al (1990) and was expressed as nmol thymine mg$^{-1}$ protein h$^{-1}$, using the molar extinction coefficient ($\varepsilon$ = 3.8 mM$^{-1}$ cm$^{-1}$) for thymine.

### Immunocytostaining and immunoblotting

Anti-human dThdPase monoclonal antibody (mouse $IgG1_{\kappa}$) was provided by the Department of Oncology, Nippon Roche Research Center (Kamakura, Japan). Mouse IgG, as a negative control, was purchased from Coulter (Florida, USA). The antibody was diluted to 2 μg ml$^{-1}$ with goat serum. Immunocytostaining was carried out using the labelled streptavidin–biotin method (Dako, Kyoto, Japan), according to the instructions of the manufacturer. The nuclei were counterstained with haematoxylin.

For immunoblotting, cultured cells were lysed in buffer containing 50 mM Tris-HCl (pH 8.8), 10% glycerol, 1% sodium dodecyl sulphate (SDS), 1% 2-mercaptoethanol and 0.0005% bromophenol blue. The lysates were electrophoresed through 10% SDS–polyacrylamide gels (20 μg per lane). The gels were transferred onto a nylon membrane (pore size 0.45 μM; Funakoshi, Tokyo, Japan). After transfer, the nylon membrane was blocked with 3% skimmed milk in phosphate-buffered saline (PBS) and probed with 2 μg ml$^{-1}$ mouse anti-human dThdPase monoclonal antibody. The kit described in the immunocytostaining method was used. Finally, 5 mg of 3,3′-diaminobenzidine in 10 ml of PBS and 3 μl of 30% hydrogen peroxide were used as substrate.

### In vitro proliferation rate

Parental PC-9, PC9-D1 and PC9-DPE2 cells were each seeded in RPMI-1640 (10% fetal calf serum) at $5 \times 10^4$ cells into six-well plates. After 4, 5, 6, 7 and 8 days, cells were released using trypsin–EDTA and counted in a Coulter counter (Coulter, FL, USA).

### Drug sensitivity test

Approximately $2 \times 10^3$ cells were seeded in each well of a 96-well plate in duplicate and cultured at 37°C in 5% carbon dioxide. After 24 h, anti-cancer agents were added, and the cells were cultured for 5 days. At that stage, the medium was removed, 20 μl of 0.5% 3-[4,5-dimethylthiazol-2-yl]-2,5-diphenyltetrazolium bromide (MTT; Sigma chemical, USA) in PBS was administered and the cells were incubated at 37°C for 4 h. Two hundred microlitres of dimethyl sulphoxide (DMSO; Wako Pure Chemical Industries Osaka, Japan) was added to solubilize the MTT formazan, and the absorbance of each well was measured (Titertek Multiskan MCC/340;Dainippon Pharmaceutical, Osaka, Japan) at 540 nm (reference absorbance at 630 nm). The effect of the drugs on cell survival was expressed as a growth ratio. The growth ratio was calculated using the following equation: ($A_{540}$ drug-treated/$A_{540}$ drug-free) × 100. The test was performed independently six times, and the mean and the s.d. of the $IC_{50}$ were calculated.

### Assessment of bystander effect

PC9-DPE2 and parental PC-9 cells were mixed in various ratios, i.e. [PC9-DPE2/(parental PC-9 + PC9-DPE2)] = 0, 0.1, 0.2, 0.3, 0.4, 0.5, 0.6, 0.7, 0.8, 0.9 and 1.0, and were seeded at $2 \times 10^3$ cells per well in 96-well plates. After 24 h, the cells were divided into two subgroups – one was untreated and the other was treated with 10 μl 5′-DFUR –, and an MTT assay was performed 5 days after the drug treatment as described above. Data were expressed as growth ratio (%) relative to drug-free controls.

To examine whether the bystander effect requires direct cell–cell contact, parental PC-9 cells were plated in the bottom chamber of 24-well culture plates, and PC9-D1 or PC9-DPE2 cells were placed in the top chamber of the membrane culture inserts (mixed cellulose ester membrane, pore size 0.45 μm; Iwaki, Chiba, Japan). The cells were incubated at 37°C in 5% carbon dioxide, and the test drugs were added into the membrane culture inserts at various concentrations 24 h later. Five days after drug treatment, the membrane culture inserts and medium were removed, 200 μl of 0.5% MTT in PBS was added, and cells were incubated at 37°C for 4 h. Finally, 1 ml of DMSO was added, and the absorbance at 540 nm was measured for each well (UV-160A;

© Cancer Research Campaign 1997

**Figure 1** Correlation between dThdPase activity and doxifluridine sensitivity in human cancer cell lines. Correlation between dThdPase activities and $1/IC_{50}$ values for doxifluridine in PC-3 (■), PC-9 (●), AGS (◆), SW480 (▲), Lovo (□), T47D (○) and Colo201 (△) cell lines was significant ($r = 0.92$, $P < 0.01$)

**Table 1** dThdPase activities of parental PC-9, PC9-D1 and PC9-DPE2 cells

| | dThdPase activity (nmol thymine $mg^{-1}$ protein $h^{-1}$) |
|---|---|
| Parental PC-9 | 28.7 ± 24.0 |
| PC9-D1 | 28.0 ± 26.7 |
| PC9-DPE2 | 1490.7 ± 276.0 |

(Brackets: PC9-D1 vs PC9-DPE2 *; Parental PC-9 vs PC9-DPE2 *)

dThdPase activity (nmol thymine $mg^{-1}$ protein $h^{-1}$) was measured spectrophotometrically. Each value represents the mean ± s.d. of seven independent experiments. *$P < 0.01$.

Shimazu, Kyoto, Japan). As control experiments, individual parental PC-9 or PC9-DPE2 cells were seeded in 24-well plates without the membrane culture inserts, and cell survival was tested in the same way as described above.

### Statistical analysis

The coefficient of correlation with dThdPase activity and 5′-DFUR sensitivity of the cell lines was calculated with Pearson's correlation coefficient, and the significance was tested with Fisher's calibration.

The differences of the enzyme activity and drug sensitivity ($IC_{50}$) were compared using the Student's $t$-test or Welch's $t$-test.

**Figure 2** Immunocytostaining of parental PC-9, PC9-D1 and PC9-DPE2 cells. Immunocytostaining was performed using mouse anti-dThdPase monoclonal antibody as described in 'Materials and methods'. The cytoplasm of PC9-DPE2 cells (**A**) was heavily stained, while that of parental PC-9 cells (**B**) and PC9-D1 cells (**C**) was weakly stained

**Table 2** Drug sensitivity ($IC_{50}$, μM) of parental PC-9, PC9-D1 and PC9-DPE2 cells

| | Doxifluridine | Tegafur | 5-FU | Cisplatin |
|---|---|---|---|---|
| Parental PC-9 | 103.5 ± 42.9 | 59.8 ± 22.7 | 3.17 ± 1.66 | 1.06 ± 0.27 |
| PC9-D1 | 94.9 ± 43.2 | 37.3 ± 16.6 | 2.24 ± 1.01 | 1.31 ± 0.42 |
| PC9-DPE2 | 0.62 ± 0.35 | 2.26 ± 1.04 | 0.39 ± 0.07 | 1.31 ± 0.53 |

(Brackets: for Doxifluridine, Tegafur and 5-FU, PC9-D1 vs PC9-DPE2 * and Parental PC-9 vs PC9-DPE2 *; for Cisplatin, ** for both)

$IC_{50}$ values were determined using the MTT assay. Each value is the mean ±s.d. of six independent experiments. *$P < 0.01$, **$P$-value not significant.

© Cancer Research Campaign 1997

**Figure 3** Western blot analysis of dThdPase in parental PC-9, PC9-D1 and PC9-DPE2 cells. Cell lysates were electrophoresed on 10% SDS–polyacrylamide gels, transferred to nylon membranes and probed with mouse anti-dThdPase monoclonal antibody as described in 'Materials and methods'. A 55 kDa protein band was detected in the lysate of PC9-DPE2 cells (lane 1), but not in those of PC9-D1 cells (lane 2) and parental PC-9 cells (lane 3)

**Figure 4** Bystander effect for doxifluridine on PC9-DPE2 cells. Parental PC-9 and PC9-DPE2 cells were co-cultured in various proportions, and the sensitivity to 5′-DFUR was examined. Treatment with 10 μM 5′-DFUR showed a clear bystander effect when the PC9-DPE2 – (parental PC–9 + PC9-DPE2) ratio was 0.1, and it is more pronounced at a ratio of 0.2 or more (*$P < 0.01$). ▮, Observed growth ratio; ▯ predicted growth ratio. Error bars represent the standard deviation

**Figure 5** Cytotoxic effect of 5′-DFUR (**A**) and tegafur (**B**) in non-contact conditions. The sensitivity to 5′-DFUR and tegafur of parental PC-9 cells that were co-cultured with PC9-DPE2 cells in non-contact (□) was significantly higher than that of parental PC-9 cells alone (■) and parental PC-9 cells which were co-cultured with PC9-D1 cells in non-contact (▲)(*$P < 0.01$). ●, PC9-DPE2 cells alone. Error bars represent the standard deviation

Differences were considered to be significant when the probability ($P$) value was <0.05.

### RESULTS

### Correlation between dThdPase activity and 5′-DFUR sensitivity of human cancer cell lines

We found a significant correlation between dThdPase activity and sensitivity to 5′-DFUR in various human cancer cell lines, including PC-3, PC-9, Colo 201, T-47D, Lovo, SW480 and AGS ($r = 0.92$, $P < 0.01$, Figure 1).

### dThdPase activity of parental PC-9, PC9-D1 and PC9-DPE2 cells

We measured the dThdPase activity of parental PC-9, PC9-D1 and PC9-DPE2 cells and confirmed that the dThdPase activity of PC9-DPE2 cells was approximately 50 times higher than that of the others (Table 1).

### Characterization of parental PC-9, PC9-D1 and PC9-DPE2 cells

Parental PC-9, PC9-D1 and PC9-DPE2 cells were immunostained using anti-human dThdPase monoclonal antibody. The cytoplasm of the PC9-DPE2 cells was heavily stained while that of the parental PC-9 and PC9-D1 cells stained weakly (Figure 2).

In the immunoblot analysis using a mouse anti-human dThdPase monoclonal antibody, a 55 kilodalton protein band was detected in the lysate of PC9-DPE2 cells, but not in that of parental PC-9 and PC9-D1 cells (Figure 3).

In order to examine whether the expression of dThdPase would influence the cell lines in vitro, the cell doubling times were assessed. Parental PC-9, PC9-D1 and PC9-DPE2 cells had a doubling time of 40.9, 40.4 and 40.3 h, respectively, with a starting culture of $5 \times 10^3$ cells. The proliferation rate of PC9-DPE2 cells was very similar to that of parental PC-9 and PC9-D1 cells.

### Drug sensitivity of parental PC-9, PC9-D1 and PC9-DPE2 cells

The sensitivity of parental PC-9, PC9-D1 and PC9-DPE2 cells to 5′-DFUR, tegafur, 5-FU and CDDP was assessed. PC9-DPE2 cells were 167, 26 and 8 times more susceptible to 5′-DFUR, tegafur and 5-FU, respectively, than parental PC-9 cells ($P$ was less than 0.01 for all three drugs). The sensitivity to CDDP among the three clones was not significantly different. (Table 2).

© Cancer Research Campaign 1997

### Bystander effect

Parental PC-9 and PC9-DPE2 cells were co-cultured in various proportions, and the sensitivity to 5′-DFUR was examined. During the treatment with 10 μM 5′-DFUR, there is a clear bystander effect at a ratio of 0.1, and it is more pronounced at 0.2 (Figure 4).

We next examined whether this bystander effect would require direct cell–cell contact. The sensitivity of parental PC-9 cells, co-cultured with PC9-DPE2 cells but not in contact, was significantly higher than that of parental PC-9 cells alone when 5′-DFUR was used at 1 μM or higher. Sensitivity to tegafur showed a similar tendency to that of 5′-DFUR, but the drug concentration required to achieve the bystander effect was ten times higher than that with 5′-DFUR (Figure 5).

### DISCUSSION

5′-DFUR and tegafur, prodrugs of 5-FU, are anti-cancer agents activated by dThdPase (Cook et al, 1979; Kramer et al, 1979; Haraguchi et al, 1993). It is generally known that human tumours have higher dThdPase activity than non-neoplastic tissues in the same organs. Consequently, 5′-DFUR and tegafur may have selective anti-tumour effects (Zimmerman et al, 1964; Fujita et al, 1983; Kono et al, 1983; Miwa et al, 1987; Yoshimura et al, 1990; Mahmoud et al, 1993). There is remarkable heterogeneity in the dThdPase activity of various tumours (Heldin et al, 1993). Tumours expressing low levels of dThdPase activity may be insensitive to 5′-DFUR and tegafur. This seems to be compatible with the present findings indicating a significant correlation between dThdPase activity and 5′-DFUR sensitivity of various human cancer cell lines.

We examined whether the sensitivity of cancer cells to 5′-DFUR and tegafur could be changed by controlling the expression of dThdPase in vitro. PC9-DPE2 cells, transfected with PD-ECGF cDNA, had a more than 50 times higher activity of dThdPase than parental PC-9 cells or than PC9-D1 cells, transfected with the pcDNA3 vector alone. The sensitivity of PC9-DPE2 cells to 5′-DFUR, tegafur and 5-FU was 167, 26 and 8 times higher, respectively, than that of parental PC-9 cells. Haraguchi et al (1993) have reported that the sensitivity of KPE-3, KB cells transfected with PD-ECGF cDNA, to 5′-DFUR and tegafur was significantly higher than that of the untransfected cells. However, the difference in sensitivity to 5-FU between KPE-3 and KB cells was not significant. Patterson et al (1995) also demonstrated that the sensitivity of MCF-7 cells transfected with dThdPase cDNA to 5-FU was not higher than that of the untransfected cells. These results are different to those obtained in this study. The increase of sensitivity to 5-FU in PC9-DPE2, however, was marginal. The enzymes that convert 5-FU to 5-fluorodoxyuridine monophosphate are generally said to be pyrimidine phosphorybosyl transferase and ribonucleotide reductase. Therefore, we cannot conclude at present whether the difference in sensitivity to 5-FU would be caused by enhanced activity of dThdPase.

In the current study on bystander effect, 10 μM 5′-DFUR yielded a minor effect with a PC9-DPE2/(parental PC-9 + PC9-DPE2) ratio of 0.1, and a sufficient effect with a ratio of 0.2 or more. We found that this bystander effect did not require cell–cell contact with 5′-DFUR (10 μM) and tegafur (100 μM). As far as we are aware, this is the first report indicating that a bystander effect for PD-ECGF/dThdPase-transfected cells does not occur via gap junctions.

Several investigators have reported a bystander effect in the HSV-TK–Ganciclovir system which is clinically useful as a gene therapy. The bystander effect in the HSV-TK – ganciclovir system has been reported to require cell–cell contact (Freeman et al, 1992, 1993*b*; Bi et al, 1993). On the other hand, it has been reported that cancer cells sensitized to cyclophosphamide and ifosfamide by transfection with the rat P-450 (CYP2B1) cDNA do not require cell–cell contact for the bystander effect (Wei et al, 1994; Chen et al, 1995). It appears that ganciclovir metabolites cannot leave the cell as they are nucleotides, while 5-FU, metabolites of 5′-DFUR and tegafur and 4-hydroxycyclophosphamide formed by CYP2B1 from cyclophosphamide readily diffuse across cell membranes. As only homologous cells are joined by gap junctions (Yamasaki, 1991; Pitts, 1994), the contact-type bystander effect may be clinically advantageous because cytotoxic metabolites would transfer to adjacent cancer cells, but not to the surrounding normal tissue. However, the contact-type bystander effect may be disadvantageous against cancerous pleuritis and peritonitis, in which cells are suspended, and against cancer tissue, in which cells are not joined by gap junctions. 5-FU converted by dThdPase can diffuse, even if gap junctions are down-regulated. Therefore, the bystander effect of the non-contact type found in this study seems more beneficial in such cases.

It has been shown that PD-ECGF(dThdPase) stimulates chemotaxis of endothelial cells in vitro and has angiogenic activity in vivo (Ishikawa et al, 1989; Finnis et al, 1993; Fidler et al, 1994; Miyadera et al, 1995; Moghddam et al, 1995; Toi et al, 1995). Consequently, it is likely that cells transfected with PD-ECGF(dThdPase) cDNA may promote surrounding tumour growth. Therefore, we consider that a transient expression, and not a stable expression vector, should be used to diminish the risk of promoting tumour growth by gene transfection.

The usefulness of the PD-ECGF – 5′-DFUR, – tegafur or – 5-FU system for the enhancement of drug sensitivity and the non-contact type bystander effect demonstrated in vitro in this study appear to be promising enough to consider in vivo investigation.

### REFERENCES

Bi WL, Parysek LM, Warnick R and Stambrook PJ (1993) In vivo evidence that metabolic cooperation is responsible for the bystander effect observed with HSV tk retroviral gene therapy. *Hum Gene Ther* **4**: 725–731

Bradford MM (1976) A rapid and sensitive method for the quantitation of microgram quantities of protein utilizing the principle of protein-dye binding. *Anal Biochem* **72**: 248–254

Chen L and Waxman DJ (1995) Intratumoral activation and enhanced chemotherapeutic effect of oxazaphosphorines following cytochrome P450 gene transfer:development of a combined chemotherapy/cancer gene therapy strategy. *Cancer Res* **55**: 581–589

Cook AF, Holman MJ, Kramer MJ and Trown PW (1979) Fluorinated pyrimidine nucleosides. 3. Synthesis and antitumor activity of a series of 5′-deoxy-5-fluoropyrimidine nucleosides. *J Med Chem* **22**: 1330–1335

Eda H, Fujimoto K, Watanabe S, Ura M, Hino A, Tanaka Y, Wada K and Ishitsuka H (1993) Cytokines induce thymidine phosphorylase expression in tumor cells and make them more susceptible to 5′-deoxy-5-fluorouridine. *Cancer Chemother Pharmacol* **32**: 333–338

Fidler IJ and Ellis LM (1994) The implications of angiogenesis for the biology and therapy of cancer metastasis. *Cell* **79**: 185–188

Finnis C, Dodsworth N, Pollitt E, Carr G and Sleep D (1993) Thymidine phosphorylase activity of platelet-derived endothelial cell growth factor is responsible for endothelial cell mitogenicity. *Eur J Biochem* **212**: 201–210

Freeman SM, Whartenby KA, Koeplin DS, Moolten FL, Abbound CN and Abraham GN (1992) Tumor regression when a fraction of the tumor mass contains the HSV-TK gene. *J Cell Biol* **16F**: 47

© Cancer Research Campaign 1997

Freeman SM and Zwiebel JA (1993*a*) Gene therapy for cancer. *Cancer Invest* **11(6)**: 676–688

Freeman SM, Abbound CN, Whartenby KA, Packman CH, Koeplin DS, Moolten FL and Abraham GN (1993*b*) The 'bystander effect': tumor regression when a fraction of tumor mass is genetically modified. *Cancer Res* **53**: 5274–5283

Fujita H, Ogawa K, Nakagawa H, Kawaguchi K, Nfakagawa H, Kawashima K, Nakagawa Y and Doi Y (1983) Pharmacokinetics of 5′-deoxy-5-fluorouridine (5′-DFUR) by oral administration. *J Jpn Soc Cancer Ther* **18**: 916–926

Furukawa T, Yoshimura A, Sumizawa, T, Haraguchi M, Akiyama S, Fukui K, Ishizawa M and Yamada Y (1992) Angiogenic factor. *Nature* **356**: 668

Haraguchi M, Furukawa T, Sumizawa T and Akiyama S (1993) Sensitivity of human KB cells expressing platelet-derived endothelial cell growth factor to pyrimidine antimetabolites. *Cancer Res* **53**: 5680–5682

Heldin NE, Usuki K, Bergh J, Westermark B and Heldin CH (1993) Differential expression of platelet-derived endothelial cell growth factor/thymidine phosphorylase in human lung carcinoma cell lines. *Br J Cancer* **68**: 708–711

Ishikawa F, Miyazono K, Hellman U, Drexler H, Wernstedt C, Hagiwara K, Usuki K, Takaku F, Risau W and Heldin CH (1989) Identification of angiogenic activity and the cloning and expression of platelet-derived endothelial cell growth factor. *Nature* **338**: 557–561

Kono A, Hara Y, Sugata S, Karube Y, Matsushima Y and Ishitsuka H (1983) Activation of 5′-deoxy-5-fluorouridine by thymidine phosphorylase in human tumors. *Chem Pharm Bull* **31**: 175–178

Kramer MJ, Trown PW, Cleeland R, Cook AF and Grunberg E (1979) 5′-deoxy-5-fluorouridine – A new orally active antitumor agent. Comparative activity with 5-fluorouracil, 2′-deoxy-5-fluorouridine and ftorafur against transplantable tumors in mice and rats. *Proc Am Assoc Cancer Res* **20**: 79

Mahmoud HK, Mustapha MK and Fardos NMN (1993) Differences in activities and substrate specificity of human and murine pyrimidine nucleoside phosphorylases: implications for chemotherapy with 5-fluoropyrimidines. *Cancer Re*ʆ **53**: 3687–3693

Miwa M, Nishimura J, Kamiyama T and Ishitsuka H (1987) Conversion of 5′-deoxyfluorouridine to 5-FU by pyrimidine nucleoside phosphorylases in normal and tumor tissues from rodents bearing tumors and cancer patients. *Jpn J Cancer Chemother* **14**: 2924–2929

Miyadera K, Sumizawa T, Haraguchi M, Yoshida H, Konstanty W, Yamada Y and Akiyama S (1995) Role of thymidine phosphorylase activity in the angiogenic effect of platelet-derived endothelial cell growth factor/thymidine phosphorylase. *Cancer Res* **55**: 1687–1690

Miyazono K, Okabe T, Urabe A, Takaku F and Heldin CH (1987) Purification and properties of an endothelial cell growth factor from humn platelets. *J Biol Chem* **262**: 4098–4103

Moghaddam A and Bicknell R (1992) Expression of platelet-derived endothelial cell growth factor in *Escherichia coli* and confirmation of its thymidine phosphorylase activity. *Biochemistry* **31**: 12141–12146

Moghaddam A, Zhang HT, Fan TPD, Hu DN, Lees VC, Turley H, Fox SB, Gatter KC, Harris AL and Bicknell R (1995) Thymidine phosphorylase is angiogenic and promotes tumor growth. *Proc Natl Acad Sci USA* **92**: 998–1002

Moolten FL and Wells JM (1990) Curability of tumors bearing herpes thymidine kinase genes transferred by retroviral vectors. *J Natl Cancer Inst* **82**: 297–300

Oldfield EH, Ram Z, Culver KW, Blaese RM, Devroom HL and Anderson WF (1993) Gene therapy for the treatment of brain tumors using intra-tumoral transduction with the thymidine kinase gene and intravenous ganciclovir. *Hum Gene Ther* **4**: 39–69

Patterson AV, Zhang H, Moghaddam A, Bicknell R, Talbot DC, Stratford IJ and Harris AL (1995) Increased sensitivity to the prodrug 5′-deoxy-5-fluorouridine and modulation of 5-fluoro-2′-deoxy-deoxyuridine sensitivity in MCF-7 cells transfected with thymidine phosphorylase. *Br J Cancer* **72**: 669–675

Pitts JD (1994) Cancer Gene Therapy: a bystander effect using the gap junctional pathway. *Mol Carcinogen* **11**: 127–130

Sumizawa T, Furukawa T, Haraguchi M, Yoshimura A, Takeyasu A, Ishizawa M, Yamada Y and Akiyama S (1993) Thymidine phosphorylase activity associated with platelet-derived endothelial cell growth factor. *J Biochem* **114**: 9–14

Suzuki S, Hongu Y, Fukazawa H, Ichihara S and Shimizu H (1980) Tissue distribution of 5′-deoxy-5-fluorouridine and derived 5-fluorouracil in tumor-bearing mice and rats. *Gann* **71**: 238–245

Toi M, Hoshina S, Taniguchi T, Yamamoto Y, Ishitsuka H and Tominaga (1995) Expression of platelet-derived endothelial cell growth factor/thymidine phosphorylase in human breast cancer. *Int J Cancer (Pred Oncol)* **64**: 79–82

Usuki K, Saras J, Waltenberger J, Miyazono K, Pierce G, Thomason A and Heldin CH (1992) Platelet-derived endothelial cell growth factor has thymidine phosphorylase activity. *Biochem Bioph Res Co* **184**: 1311–1316

Wei MX, Tamiya T, Chase M, Boviatsis EJ, Chang TKH, Kowall NW, Hochberg FH, Waxman DJ, Breakefield XO and Chiocca EA (1994) Experimental tumor therapy in mice using the cyclophosphamide-activating cytochrome P450 2B1 gene. *Hum Gene Ther* **5**: 969–978

Yamasaki H (1991) Aberrant expression and function of gap junctions during carcinogenesis. *Environ Health Persp* **93**: 191–197

Yoshimura A, Kuwazuru Y, Furukawa T, Yoshika H, Yamada K and Akiyama S (1990) Purification and tissue distribution of human thymidine phosphorylase: high expression in lymphocytes, reticulocytes and tumors. *Biochim Biophys Acta* **1034**: 107–113

Zimmerman M and Seidenberg J (1964) Deoxyribosyl transfer. Thymidine phosphporylse and nucleoside deoxyribosyltranferase in normal and malignant tissue. *J Biol Chem* **230**: 2618–2621

© Cancer Research Campaign 1997